# Multisectoral collaboration on violence against women and children in Iraq, Jordan, and Lebanon: A comparative qualitative study

**DOI:** 10.1371/journal.pgph.0007014

**Published:** 2026-08-21

**Authors:** Mervat Alhaffar, Nour Horanieh, Noha Elsebaie, Dechol Ramazan, Alessandra Guedes, Loraine J. Bacchus, Ismahan Ferhat, Suzan Kasht, Nisrine Tawily, Junita Upadhyay, Carlos Javier Aguilar, Manuela Colombini

**Affiliations:** 1 Department of Infectious Disease Epidemiology and International Health, Faculty of Epidemiology and Population health, London School of Hygiene & Tropical Medicine, London, United Kingdom; 2 Syria Research Group (SyRG), co-hosted by the London School of Hygiene & Tropical Medicine, London, United Kingdom; Saw Swee Hock School of Public Health, Singapore, Singapore; 3 Department of Global Health and Development, Faculty of Public Health and Policy, London School of Hygiene & Tropical Medicine, London, United Kingdom; 4 Department of Family and Community Medicine, College of Medicine, King Saud University, Riyadh, Saudi Arabia; 5 Kroc Institute for International Peace Studies, Keough School of Global Affairs, University of Notre Dame, Notre Dame, Indiana, United States of America; 6 Independent consultant, Erbil, Iraq; 7 UNICEF Office of Strategy and Evidence – Innocenti, Florence, Italy; 8 UNICEF Middle East and North Africa Regional Office, Amman, Jordan; 9 UNICEF Jordan Country Office, Amman, Jordan; 10 UNICEF Lebanon Country Office, Beirut, Lebanon; 11 UNICEF Iraq Country Office, Baghdad, Iraq; University of Antwerp, BELGIUM

## Abstract

Increasing global evidence highlights the significant intersections between violence against women (VAW) and violence against children (VAC), emphasising the need for collaboration among actors addressing VAW and VAC. However, tensions regarding prioritising women’s and children’s needs have led to fragmented approaches, in addition to knowledge gaps regarding effective collaborative efforts. This study aims to assess how the coordination between VAW and VAC programmes is conceptualised and operationalised in Iraq, Jordan, and Lebanon. Specifically, it seeks to identify a) how VAW and VAC collaboration is understood, b) challenges to its implementation, and c) recommendations for strengthening it. We conducted a situation analysis utilising document review and qualitative interviews. A policy document analysis was conducted across the study countries. Thirty key informant interviews were conducted with stakeholders selected based on their experience in VAW and/or VAC work. Data were analysed thematically. Collaboration between efforts to address VAW and VAC was frequently described at the policy level through integrated, multi-sectoral family protection approaches, particularly in Lebanon and Jordan. Overall, few programmes and services adopted a coordinated approach to VAC and VAW, with varying concept understanding and quality across the three countries. A key cross-country finding was that VAC is often positioned as a more socially and politically acceptable entry point for addressing family violence, allowing actors to indirectly engage with VAW in cultural contexts where gender norms constrain specifically addressing VAW. Recommended opportunities for simultaneously preventing VAC and VAW included community-based youth programs, parenting initiatives, and involvement of cultural and religious leaders. The study highlights existing, albeit limited, coordinated approaches to addressing VAW and VAC in the three settings, however, their implementation is constrained by structural and systemic barriers. Given ongoing resource limitations,identifying interventions that can safely address both VAW and VAC outcomes is critical, when appropriate and safe to do so.

## Introduction

Violence against women (VAW) and violence against children (VAC) are major global health concerns, important human rights violations, and significant barriers to sustainable development [1]. One in three women globally experiences intimate partner violence (IPV), leading to adverse physical, mental and reproductive health consequences [2–5]. Half of children aged 2–17 years experience some form of violence each year, often perpetrated by parents or caregivers [6]. Nearly 75% of young children have experienced physical or psychological violence from a caregiver or a parent [7]. Experiencing these forms of violence or witnessing IPV during childhood can have profound implications for children’s physical and mental health, educational attainment, and social development [8].

Evidence has shown associations between IPV and VAC, especially in low-income and middle-income countries (LMICs) [9,10]. In many cases, IPV and VAC occur within the same household. VAW and VAC intersect in six areas: common risk factors (such as gender inequality, alcohol and drug misuse, and inadequate legal protections), cultural norms that normalise violence, overlapping and intensifying impacts (such as poor mental, physical, and reproductive health, and multiple victimisations), intergenerational transmission, co-occurrence of IPV and VAC within the same family, and greater susceptibility during adolescence [11]. Both IPV and VAC are also associated with adverse mental, physical, and sexual and reproductive health outcomes [12]. In addition, early exposure to violence is linked to later victimisation among women and perpetration among men, reinforcing intergenerational cycles of violence [13]. This has prompted calls for comprehensive family-centred services [10], which in these contexts typically involve coordinated, multi-sectoral interventions, including integrated case management and referral pathways to address both IPV and VAC within households to prevent the transmission of violence across generations and achieve more sustainable outcomes. Despite increasing advocacy for joint prevention efforts and integrated support systems for women and their children, the absence of clear collaborative frameworks has led to uncoordinated and uneven responses [11] with VAW and VAC services often being fragmented and siloed (i.e., characterised by limited coordination and communication between relevant actors and services). In this paper, we use multisectoral collaboration to describe how VAW and VAC actors, policies and services work across sectors and institutional mandates, including through shared policies, referral pathways, coordination mechanisms and day-to-day working relationships. Greater recognition of the ways in which VAW and VAC intersect, and of their overlapping risk factors, has prompted researchers and practitioners to adopt collaborative approaches aiming for better outcomes for women and children [11,14]. In a recent systematic review of interventions that jointly address IPV and VAC by parents/caregivers, Bacchus and Colombini et al. (2024) found that most interventions focused on prevention, while fewer on response, and only a handful (mainly health service interventions) addressed both IPV and VAC. Interventions that simultaneously prevent IPV and VAC tended to work through shared mechanisms, such as improving couple and family communication, enhancing non-violent conflict resolution skills, and challenging harmful gender norms. However, most evidence on coordinated interventions derives from high-income countries for response-oriented interventions but not prevention-oriented ones, and from some African contexts for prevention-focused approaches. Evidence from the Middle East and North Africa region remains limited [1].

In the Eastern Mediterranean region, the IPV prevalence is 27% [2,5], while for VAC in the home it is 88% on average for children under 5 years old [15]. The VAW & VAC landscape in the region is particularly complex due to region-specific socio-political dynamics including occupation, oppression, ongoing/protected conflicts and patriarchal norms [16–18]. These factors intersect, underscoring the complexity of addressing VAW and VAC in the region, where political, cultural, religious, economic, and structural forces shape and sustain multiple forms of violence [19].

Violence against women and children is pervasive across Iraq, Jordan, and Lebanon, particularly IPV (20% in Iraq, 12% in Jordan and 40% in Lebanon) [5] and violent child discipline (57% in Lebanon [20] and 81% in Iraq [21]). While these countries have introduced some protective legislations (e.g. Kurdistan’s 2011 Domestic Violence Act [22], Jordan’s 2017 Protection from Domestic Violence Act and Lebanon’s Laws 422/2002 and 293/2014), significant gaps persist. Iraq still lacks a federal domestic violence (DV) law (Domestic violence (DV) is a broader term encompassing violence within the household or family. Intimate partner violence (IPV) refers specifically to violence occurring within an intimate relationship. Although the terms are sometimes used interchangeably in policy and practice, IPV is a subset of DV. Gender-based violence (GBV) refers to harmful acts directed at individuals based on their gender and rooted in unequal power relations; it encompasses a range of abuses, including but not limited to IPV), Jordan does not recognize rape occurring within marriage, and enforcement across all three contexts (Iraq, Jordan and Lebanon) remains weak, leaving women and children partially protected from abuse. Additional information on prevalence and regulatory frameworks is presented in Table 1 below. Despite efforts to address IPV and VAC through national policies and legal frameworks, significant gaps in implementation, reporting, and cultural acceptance persist. The Iraqi Penal Code includes provisions addressing gender-based violence (GBV), but societal norms and prevailing legal interpretations often undermine their protective intent. In Jordan, for example social norms commonly reinforce the view that IPV and other forms of violence within the home are private matters [26]. Similarly, physical discipline of children is widely accepted, with parents and family members being among the main aggressors [27]. Furthermore, each country has distinct yet interconnected political landscape that shape their responses to VAW and VAC. For instance, the situation in Lebanon is complex as the country grapples with multiple internal crises and the spillover effects of neighbouring conflicts, all of which contribute to a state of economic and social instability. These compounding crises have severely increased violence against women and children, as poverty and economic instability heighten the risk of violence in the home and beyond [28,29]. These contexts differences matter because they shape how much space there is for VAW and VAC actors to work together, including through policies, referral pathways and everyday working relationships.

**Table 1:** VAW and VAC prevalence and regulatory frameworks in Iraq, Jordan and Lebanon.

Country	Prevalence of IPV	Prevalence of VAC	VAW and VAC Regulatory frameworks
**Iraq**	20% of women (15–49y) experienced IPV in their lifetime [5]	• 81% of children aged (1-14y) face violent discipline, with 31% of them experiencing severe physical punishment [21]	• Iraqi Constitution (2005) prohibits family violence [23] • Kurdistan Region of Iraq (KRI) has made significant legal advancements, including excluding wives from “disciplining” provisions and enacting the Domestic Violence Act No. 8 of 2011 • Iraq lacks specific federal legislation criminalising domestic violence
**Jordan**	12% of women experienced spousal physical, sexual or emotional violence [5]	• 75% of children aged (1–14y) experienced at least one form of violence by parents/caregivers [24] • 12% of young girls(15–17y) are married [25]	• National Framework for Protection for family violence (2017) • Gaps in legal protection from marital rape
**Lebanon**	40% of women aged 15–49 experienced physical and/or sexual IPV [5]	• 57% of children experience violent discipline [20] • Increased maltreatment reported during crises	• Laws 422/2002 and 293/2014 aimed at protecting women and children from violence

## Methods

### Ethics statement

Ethical approval for the study was obtained from the London School of Hygiene & Tropical Medicine Ethics Committee (Ref: 28846). The study adhered to ethical and safety protocols for qualitative interviews with key informants, including informed consent, confidentiality and anonymity, and ensuring participant safety. With support from the UNICEF Jordan country office, local approval was obtained to interview Ministry of Health staff. It was not possible to obtain formal ethical approval in all three countries due to the absence of national ethics committees for studies that do not involve direct service users or patients. In such cases, UNICEF country offices facilitated necessary permissions with national institutions and oversight to ensure adherence to local ethical standards.

### Study design and methodological approach

This study used a qualitative approach comprising 1) documentary analysis of VAW and VAC policies in Iraq, Jordan, and Lebanon; and 2) semi-structured interviews with key informants working within the VAW and VAC sectors in the three countries.

The aim of the study was to explore collaboration between VAW and VAC fields as a spectrum of coordination mechanisms, rather than as synonymous with full structural integration, reflecting how collaboration was understood across the study contexts. The main objectives were to: a) identify existing policies, action plans, programmes that address both violence against women and violence against children; and b) examine challenges and opportunities affecting collaborative approaches between the two fields.

#### Documentary analysis.

A desk-based document analysis was conducted by MA, MC, and NE to systematically identify and review publicly available national laws, regulations, policies, protocols, and grey literature addressing both VAW and VAC in the three study countries. Keywords searches were conducted across government websites, NGOs, Google, Google Scholar, and UN agencies databases in both English and Arabic. Only documents published from 2010 onward were included,and only if they referenced both VAW and VAC across multiple sectors (including health, education, justice, and social services). Individual VAW and VAC policy documents were also reviewed to assess whether they included areas of overlap, shared mandates, referral pathways or coordination mechanisms between the two fields. Documents were excluded if they focused solely on one form of violence, or specifically and exclusively on a narrow subgroup or service setting without wider relevance to national VAW and VAC policy or coordination. For Iraq, this criterion was applied to both federal and Kurdistan Regional Government documents. The final sample (n = 18) was organised into three categories: i) national legal and policy frameworks; ii) sector-specific guidelines and protocols; and iii) other relevant reports. For each country, we developed a structured matrix to extract relevant information on multi-sectoral policies, services and practices addressing VAW and VAC together, and to identify provisions that might unintentionally exacerbate either form of violence. We then compared the matrices across Iraq, Jordan and Lebanon to identify common patterns and country-specific differences in how VAW and VAC were addressed together, and where collaboration was enabled, limited or unclear. Referral pathways and joint case-management arrangements were included in the extraction matrix, with gaps identified through the content analysis rather than through a formal checklist or farmework. A content analysis approach, based on Graneheim and Lundman [30,31], guided our analysis. More detailed information is available in the country reports [32–34].

#### Semi-structured interviews.

***Sampling and recruitment*:** Eligible participants were national-level stakeholders from governmental or non-governmental organisations (NGO) with experience in addressing VAW and/or VAC (see Table 2). Thirty participants were purposively selected with support from UNICEF regional and country offices, based on their professional role. Following introductory emails from UNICEF colleagues, lead author MA followed up with study information sheet and consent forms.As recruitment was supported by UNICEF, and through its networks, participants were more likely to be already known to UNICEF or involved in national-level coordination and donor-supported VAW and VAC work.

**Table 2:** Participants in Iraq, Jordan, and Lebanon.

Country/Sector	Iraq	Jordan	Lebanon	Total
Government Actors	5	5	5	15
NGO Staff	4	3	6	13
Semi-governmental organisations (operate with a degree of independence, but are partially funded/overseen by the government)	0	2	0	2
Total Participants	9	10	11	30

***Data collection*:** Thirty key informant interviews were conducted between July and November 2023 across the three countries. A semi-structured topic guide, developed in English and translated into Arabic, explored joint programmes and services where VAW and VAC intersected, barriers and facilitators to implementing coordinated approaches, and opportunities for increased coordination. After obtaining written informed consent, MA conducted 11 interviews (8 in-person in Beirut and 3 online via zoom) in Lebanon; and 12 interviews in Jordan (10 in-person in Amman and Irbid, of which 2 were held in pairs, and 2 via zoom). All interviews were conducted in Arabic, lasted approximately 40–60 minutes, and were recorded (except for one where the participant did not consent and extensive notes were taken instead). In Iraq, nine interviews were conducted by DR (who was trained on the topic guide and had prior experience conducting qualitative interviews) in Erbil & Baghdad, with one additional interview conducted online by MA over Zoom. Owing to the sensitive nature of the topic, only the online interview was recorded, while extensive summary notes were taken for all other interviews. In Iraq, four interviews were conducted in Arabic, five in Kurdish and one in English (online). All in-person interviews were conducted in locations chosen by the participants.

***Data analysis*:** Interviews were transcribed into Arabic with four transcripts translated into English to enable shared analysis across the research team. We drew on Braun and Clark’s [35] thematic analysis following its iterative stages of familiarisation, coding, theme development, review and refinement, to explore participants’ views on collaboration between VAW and VAC initiatives; factors facilitating or hindering collaboration; and recommendations for improving collaborative approaches.

Coding was initially carried out in Arabic by MA, NH, and NE. Codes were generated both inductively and deductively. Deductive coding was guided by the study’s research questions and relevant literature on VAW–VAC collaboration, and inductive coding allowing new patterns to emerge from participants’ accounts. To enhance the reliability of coding, MA and MC independently annotated four transcripts to identify common patterns and themes. These initial insights were discussed during three country-specific data analysis workshops, conducted in English, to refine themes, reconcile coding discrepancies, ensure consistency in interpretation, and help reach consensus on a tentative coding frame, which was refined iteratively as additional transcripts were coded. Once finalised, the coding framework was applied to the remaining transcripts. Data were systematically organised into Excel matrices, structured by theme, country, and participant characteristics to support cross-country comparisons and across stakeholder groups, and institutional settings. To ensure accuracy and preserve meaning, illustrative quotes were translated into English by bilingual co-authors. Three online validation workshops were subsequently held with UNICEF regional and the three country offices to ensure the validity and contextual relevance of the findings and to refine the recommendations.

Working in both Arabic and English, iteratively refining the code book, and engaging UNICEF country offices helped mitigate linguistic and cultural influences and researcher bias, thereby enhancing the rigor and credibility of the analysis.

### Inclusivity in global research

Additional information regarding the ethical, cultural, and scientific considerations specific to inclusivity in global research is included in the Supporting Information (S1 Checklist).

## Results

This section first summarises key findings from the documentary analysis, followed by results from the qualitative interviews.

### Document analysis results

#### Policies addressing VAW and VAC coordination.

Our analysis of 18 policy and guidance documents indicates that all three countries have national regulatory instruments that address VAW and VAC, although their depth and coordination mechanisms vary. Lebanon and Jordan have more comprehensive policy systems (e.g., national strategies, multi-sectoral action plans, and sector-specific guidelines) that explicitly promote integrated prevention and response services for VAW and child protection. Lebanon’s Ministry of Social Affairs’ (MoSA) plan and Lebanon’s QUDWA social and behaviour-change plan both acknowledge shared drivers such as harmful gender and social norms [20]. Iraq has a patchwork of provisions, including the Kurdistan Regional Government’s 2011 Domestic-Violence Act and two national VAW strategies [22], which call for coordination but offer limited operational detail.

Across all three countries, the documents primarily focused on protection from family violence, including GBV, DV, and child protection (CP). Documents from Lebanon and Jordan articulate a multi-sectoral approach to addressing violence and integrated service provision, while those from Iraq recognise the need for coordination. However, despite policy-level commitments to multisectoral approaches, guidance on how different ministries and other stakeholders should work together remains limited across all three settings. At the service level, one notable exception is Lebanon’s 2022 Child Protection Guidelines for Health Providers, which link IPV screening with broader household-violence risk assessment. In Jordan, the School-Environment Strategy and Child-Protection Guidelines for Health Providers similarly reflect integrated thinking across the education and health sectors. However, these documents stop short of providing practical guidance on referral pathways or joint case-management protocols. Table 3 summaries key VAW and VAC policy documents we reviewed in each country.

**Table 3:** Summary of key VAW and VAC policies in Iraq, Jordan, and Lebanon.

Country	Document title	Main aim	Mentions of VAW and VAC
**Iraq & KRI**	Iraqi Penal Code 111/1969	The penal code permits “discipline” of wives and children within legal/custom limits (Art. 41). Minor assault causing no visible injury is punishable (Art. 415), and “honour” can mitigate family-violence crimes, treated differently from murder.	•Domestic violence against women and child corporal punishment by parents.
	KRI Domestic Violence Act No 8 2011	This Act discusses the prohibition of DV within families and provides examples of acts considered as DV. It uses a very broad understanding of acts considered as DV to also include child marriage and female genital mutilation. It also mandates for establishment of government-run shelters for women.	•VAW: forced marriage, child marriage, FGM, forced divorce, disowning family members, forcing prostitution, causing suicide or abortion due to DV, physical and psychological abuse. •VAC: preventing family members from working or going to school, forcing children to work or beg, beating children and family members.
	National Strategy to Confront VAW in Kurdistan (2017-2027)	This strategy focuses on VAWG and is divided across: 1.Legal: Eliminate legal discrimination and provide legal protection 2.Prevention: Raising awareness of VAW 3.Protection: Supporting and protecting 4.Care: Improving services for survivors	•VAW forms: child and forced marriages; honor killings; DV, FGM and burning. •VAC only mentions around forced marriage of teenage girls (girls being married at 15 & lower).
	National Strategy to Combat VAWG (2018-2030) – Federal level	This strategy emphasizes importance of combating violence against women and children to ensure safety, well-being, and empowerment. It identifies gaps within legal framework and ensures an effective coordination and monitoring mechanism for the Strategy.	•Main focus is on VAW: physical, psychological, economic and sexual violence; FGM. •Though also mentions VAC: child maltreatment including mistreatment at home and at school, in juvenile facilities.
**Jordan**	National Framework for Family Protection from Violence (2016)	This framework provides national reference protecting families from violence, mentions survivor-centered and multi-sectoral approach. It sets out roles & responsibilities for coordination.	•Addresses women & children. •Only defines family violence. •Mentions survivor-centered and multisectoral approach for GBV, but does not detail how services could coordinate to jointly address VAW and VAC.
	Executive Plan for the National Priorities for Strengthening the Response to GBV, DV and CP (2021-23)	This plan addresses VAW in an integrated way, highlights key interventions for each sector (health, social services, police & justice), and mentions multi-sectoral coordination.	•Works with both women and children. •Has created a hotline and reporting mechanisms (an app) for VAW and VAC.
	The National Strategy for Women 2020–2025	This is a national roadmap on gender equality and includes key pillars that the Jordanian Government has identified as key priorities. Gender-based violence is one of the four pillars of the strategy.	•Recognises GBV affects women and girls, including within and beyond the family (e.g., sexual exploitation). •On access to justice, calls for greater equity within the family and safeguarding the child’s best interests.
	Action Plan for the National Strategy for Women 2023–2025	This action plan lays out key interventions for collaboration between VAW and VAC.	•Includes Strategic Goal 2: Women and girls live free from all forms of violence. •New family-violence initiatives: strengthen prevention mechanisms and develop an early-detection system for GBV, family violence, and child protection cases (not yet operational).
	Standard Operating Procedures (SOPs) for the Prevention of and Response to Cases of Violence (2018)	This document lays the foundations of multi-sectoral coordination by: i) establishing a common understanding between al specialists and workers in the field of protection; and ii) defines roles and responsibilities.	•GBV, DV and CP from violence, exploitation and neglect.
**Lebanon**	National Strategy to Combat Violence against Women and Girls (2019-29)	This strategy aims to raise awareness on the negative effects of various forms of VAW on survivors, families and societies. Only Strategic Objectives 1 and 3 explicitly mention protecting/responding to women and girls and women/children survivors/victims, but neither explains how to address VAW and VAC together.	•All GBV against women and children.
	Strategic Plan on the Protection of Women and Children (2020-2027)	This document offers a road map for the protection of women and children and the strengthening of the national system for the prevention and response to VAW and VAC.	•VAW and VAC (e.g. Sexual Violence (SV), violent discipline). •Child marriage, Violence against women and girls (VAWG) and boys.
	QUDWA-National Social and Behavioural Change and Communication (SBCC) plan* *Under Objective 4 of the Strategic Plan for the Protection of Women and Children	This plan aims to address the root causes of harmful against girls, boys, and women such as harmful cultural and social norms, limited awareness and community dialogue, and weak community protection and support systems.	•Addresses GBV, VAW, and VAC.
	Lebanon Crisis Response Plans (2022–2023 and 2023)	These plans are a key milestone to strengthen protection of women, children and boys from sexual and gender-based violence (SGBV) in conflict. The 2023 plan sets coordinated actions across sectors (health, education, protection) and highlights adolescent violence, especially child marriage.	•Gender-based violence, sexual exploitation and abuse, and child safeguarding.
	Strategy for Protecting Students in The School Environment (2017)	This strategy aims to protect students from violence and outlines prevention activities on any form of violence within or outside the school, including DV.	•SV in and outside school, including domestic violence. •It mentions ‘family dysfunction’ as a risk for children witnessing DV.
	Child Protection Guidelines for Health Providers (2022)	These guidelines mainly focus on VAC and also explain how to screen and address for DV/GBV against women.	•VAC but also include DV.

### Semi-structured interviews results

Findings from the key informant interviews are organised around three main themes: i) how collaboration between VAW and VAC initiatives is framed and understood, ii) challenges to VAW and VAC collaborative approaches, and iii) recommendations for strengthening VAW and VAC collaboration.

#### How VAW and VAC collaboration is framed and understood?

The findings highlight broad agreement across the three countries that outreach and prevention programmes for VAW and VAC must be coordinated, although participants framed VAW and VAC coordination differently. Nearly all participants from Lebanon and Jordan emphasised the deep interconnections between VAW and VAC, stressing that one cannot be effectively addressed without addressing the other. Hence, they described coordination between VAW and VAC outreach and prevention programmes not simply as beneficial, but as necessary for effective response.

*“For us, there is a lot of intersection* [between VAC/VAW] *and these issues are not separated.*” [L1, government actor, Lebanon]

*“According to my experience and presence on the ground, whenever there is a gender-based violence concern, there is always a child protection risk.”* [L8, NGO staff, Lebanon]

“*It’s true that we are specialised in child [protection] only, that’s our vision, mission and objectives, but we can’t just work on fixing the child’s problem and forget about the women!”* [L6, NGO staff, Lebanon]

The NGO workers reflected on the limitations of separating VAW and VAC responses, emphasising that violence cannot be addressed by focusing on children alone. In contexts where VAC is present, mothers are also likely experiencing violence, which requires inclusion in household-level protection responses. Similarly in Iraq, some women-based NGOs supported integrated approaches to domestic violence affecting women and children in the same household, stressing the importance of safeguarding both women’s and children’s safety.

Interestingly, child marriage emerged as the clearest example through which participants framed the overlap between VAW and VAC. It was used to illustrate how certain issues do not fit neatly within either sector, as girls are simultaneously treated as children and women depending on legislations, norms and policies. Several participants across all countries criticised contradictory laws, for example, defining a child as someone under 18 while allowing marriage at 15 in Jordan. These discrepancies between the legal age of consent to marry and the age of majority leave adolescents unprotected, leading many participants to stress the need for interdisciplinary collaboration. Moreover, a few participants recognised child marriage not only as a form of violence against girls, but also as a significant risk factor for intimate partner violence, as girls married at a young age experience heightened power imbalances, adverse health impacts, increased risk of intergenerational cycles of violence, social isolation, and limited access to support services [36].

Most participants underscored the influence of social norms, religious and cultural values, tribal customs, and legislation in shaping communities’ responses to VAW and VAC as a coordinated one. Many called for family-centred services that respect and prioritise the family unit (The concept of family in the studied region is context-dependent and may encompass the nuclear family, the extended family, and, in some settings, wider kinship or tribal networks. Study participants, when using the term “family”, mainly referred to the nuclear family and, where relevant, the extended family (e.g., grandparents, aunts, uncles, and cousins)) and included the protection of both women and children. However, in line with efforts to preserve the family unit, some participants in Jordan and Lebanon described the reconciliation processes in cases of IPV, usually facilitated through signed pledges or contractual agreements between spouses by the Family Protection Department. While some participants viewed reconciliation as a potential mechanism for resolving family conflict, many also said it could allow violence to persist or escalate, a concern which is increasingly substantiated by the evidence.

*“In the overall picture, I deal with the family as a unit, as a whole, not dividing it into parts. I address any issues within the family, whether it’s with the father, the mother, or the children […] the intervention plan I would build is aimed at the family as a whole, emphasizing the need for cohesion among its members.”* [J7, government actor, Jordan]

*“When dealing with gender-based violence risks or cases, concentrating solely on GBV without addressing the needs of the child proves inadequate.”* [L8, NGO staff, Lebanon]

A repeated justification for need for VAW and VAC collaboration was to ensure logistical efficiency. Participants highlighted how coordination can and decreased redundancy and overlap, as well as unnecessary costs while allowing service providers to spend more time assessing risks within families, especially when perpetrators were family members.

*“It* [collaborate approach] *helps the family unity and resilience and I think this is the most important reason to have this approach, however I am very aware that sometimes the perpetrators could be within the family so* [for] *these cases we have been using mobile outreach* [and] *working carefully to ensure our service is safe. That can take a longer time, but it helps address these issues and reduce the risks in the family from all angles.”* [I9, NGO staff, Iraq]

At the same time, some participants valued the importance of maintaining specialist expertise in VAW and VAC to ensure tailored and appropriate support.

*“It is not a wrong thing having organisations working only on child protection or only on woman protection. However, it is all connected and we [institutions] complement each other.”* [L10, government actor, Lebanon]

This perspective highlights a key tension in how collaboration was conceptualised. While some participants recognised the value of specialised expertise for VAW and VAC, they also framed these as interdependent rather than separate domains of practice. Hence, collaboration was framed less as full integration and more as complementarity, with actors coordinating and engaging jointly rather than working in isolation.

Some government and NGO participants cautioned that focusing exclusively on child protection while neglecting the status of women in the family - or vice versa - was problematic as the two are deeply intertwined. They mentioned examples such as child marriage, children witnessing IPV even if they are not directly abused, and in some cases, children experiencing violence from mothers who are themselves victims of domestic abuse.

*“From my perspective […] if the mother is facing violence, then it will affect the child and if the child is facing violence, it’s either that she is abusing her child or she’s also facing abuse by the perpetrator alongside her child, so it’s linked, how can you detach them from one another?”* [L9, government actor, Lebanon]

Several participants noted that children witnessing violence experience psychological distress, emotional harm and academic difficulties, reinforcing the need for joint responses targeting shared risk factors.

#### Challenges to VAW and VAC collaborative approaches.

Across all three countries, participants agreed that coordination between VAW and VAC services was hampered by a mix of structural, financial, service-provision and cultural barriers, though each country context places a different emphasis: participants in Jordan dwelt on patriarchal norms, in Lebanon on political paralysis, and in Iraq on resource constraints.

***Structural barriers***: Structural barriers were particularly prominent and refer here to systemic constraints embedded within legal, policy, and organisational frameworks that shape how services are governed and delivered [37]. All participants shared frustration with the weak legal and governance framework, including the lack of consensus on VAW and VAC definitions across ministries; parallel religious (Lebanon) and tribal rules (Jordan) that hinder the application of the civil laws in courts; and inconsistent mandatory reporting rules. These inconsistencies included variation in institutional guidelines, consent requirements for reporting, and the absence of protective measures for those who report abuse.

*“There are times when the mother do not give consent to talk about VAC because she’s afraid of what the father might do to the kids but there are other times in CP work where the problem* [abuse] *with the children reflects bigger issues that the mother does not want to talk about, so she’ll allow us to talk to the children and to treat their symptoms but not to work with her [...] and usually in those cases she’s also abused.”* [L5, NGO staff, Lebanon]

A key barrier was the lack of training on collaborative approaches and limited understanding of different forms of violence among specialised service providers. While NGOs and government workers made individual efforts to create guidelines and manuals for frontline staff, participants remained frustrated by the absence of a clear national vision and roadmap to address violence.

*“Critical to our team’s development is training on case management related to gender-based violence and the nuanced handling of survivors. Equally crucial is training on general principles of child protection and the provision of child protection services.”* [I3, government actor, Iraq]

In Lebanon, a major barrier was the absence of a functioning government at the time of data collection, leaving participants to work with limited capacity, oversight, and accountability. In both Lebanon and Jordan, the Syrian conflict shaped national priorities. In Lebanon, the focus on refugees was reported to sometimes sideline programmes for local populations, although it also contributed to clearer legal frameworks on violence. In Jordan, participants highlighted child marriage in refugee camps as an increasing concern with perceived spillover effect on the host population.

***Funding barriers***: International donors often require agencies to align their programmes strictly within either GBV or child-protection frameworks, reinforcing institutional silos and limiting integrated approaches to VAW and VAC. These funding conditions shape the organisational mandates and can constrain the possibility of a collaborative approach to the two types of violence. A Lebanese NGO worker summed up the dilemma:

*“I told them* [UNICEF officials] “*we will work on CP and if we don’t work with you, we will work with someone else”, and we did work with someone else [...] for me, when you enter a household, you’re entering a unit […] if the mom is facing violence then the child is not receiving their needs, we’re talking about emotional and physical needs, because no matter how much she hides it* [violence] *and no matter how much she prioritises her kids, and stays for them, something got broken in her.”* [L5, NGO staff, Lebanon]

Local organisations reported pressure to align with international donors’ priorities, creating tensions with local needs and clashes with local laws and cultural contexts. Furthermore, in Lebanon, participants highlighted that limited budgets for VAW and VAC sectors were diverted to urgent health crises like COVID-19 and cholera, while political turmoil further deepened funding shortages, service fragmentation, and human resource gaps. Many programmes relied on undertrained volunteers and individual efforts, raising concerns about quality of care and sustainability. Similar to Lebanon, participants from Iraq and Jordan, identified the limited resources and budgets as barriers to collaborative approaches. It is important to note that the funding landscape has changed since the data collection period.

“*Government institutions are incapable of taking action due to the ongoing economic crisis, and they believe that donors are unlikely to provide further funding.”* [L4, NGO staff, Lebanon]

***Service-provision barriers***: Poor data systems, overlapping ministries’ mandates, siloed working practices, and the lengthy bureaucratic policy approvals further slowed coordinated case management in all three countries. In Iraq, NGO workers perceived health providers as having limited skills or flexibility to meet survivors’ needs, prompting NGOs to manage cases independently whenever possible.

“*From our level at the government, it’s a bit complicated because each government institute is bounded by a set of requirements from the council of ministries or from the ministries. Any collaboration for the government institutes needs to be discussed at the higher level with the key persons at the ministry levels or the council of ministries directly so then it can get down to the ministries through different directorates*”. [I7, government actor, Iraq]

Participants in Lebanon and Jordan highlighted issues in shelter regulations, where family shelters exclude boys over twelve creating additional challenges for affected mothers seeking refuge and forcing them back to their abusers’ homes.

*“The safe shelters have many restrictions. Meaning, if the victim has sons who are above 12 years old, they won’t be admitted*.” [L4, NGO staff, Lebanon]

***Cultural barriers***: In Jordan and Lebanon, participants highlighted some of the prevailing societal norm of ‘*ayb*’(shame-based culture) and male authority, as major barriers to reporting violence, for fear of facing social ostracism and sanction.

Other cultural barriers were described in relation to intruding *Western* influences on local contexts. For example, in Lebanon, two participants described scepticism and negative perceptions among male community members who viewed VAW programmes as being led by “*liberal women”.* In Jordan, participants identified several barriers to addressing VAW cases including the influence of tribal rules, fear of imposing “*Western/liberal notions of women’s rights”,* and prevailing beliefs that parents have the right to discipline children even by using violence. Yet, many participants argued that packaging VAW services within child protection or “*family protection*” programmes deemed to be more socially acceptable and improve access.

*“Having women services* [packaged] *within the child protection services destigmatises the survivors of GBV and also makes discreet and better access for those activities. For example, by providing MHPSS* [Mental Health and Psychosocial Support] *services for children and adolescent girl specific programming, we were able to work with the families and provide safe access, like one-stop shop where the entire family can have access to the support services*.” [I9, NGO staff, Iraq]

Taken together, these findings show that collaborative approaches are challenging, not because stakeholders doubt its value, but because legal clarity, stable funding, service capacity and social acceptance have yet to converge in any of the three countries.

#### Recommendations for strengthening VAW and VAC collaboration.

Participants offered concrete recommendations to strengthen collaboration between VAW and VAC initiatives. Culturally, participants urged closer engagement with community and religious leaders, as well as greater involvement of men, to build acceptance and communicate the harms of VAW for children, the family unit and the society as a whole. They further highlighted the importance of addressing harmful social norms that perpetuate violence and emphasised the need for coordinated engagement with families, schools, religious leaders, and media to promote positive social norms and end all forms of violence.

*“You work with the community as a whole, you talk about protecting children and women so people won’t think it’s crude or that you’re encouraging women to be against men [...] Working with religious leaders* [is important] *even though many organisations distance themselves from them which I understand because I believe that we have many issues that we disagree, but we need to be careful so that we do not make unnecessary enemies […] and talk about the issues we actually agree on”* [L5, NGO staff, Lebanon]

Capacity-building was a consistent priority. Almost all participants across the three countries strongly emphasised the need for joint training initiatives to strengthen collaboration between VAW and VAC actors. Such training was seen as a way to harmonise approaches, improve coordination, and foster a shared understanding among professionals when responding to cases, particularly those involving adolescents.

Equally important, participants highlighted the need to support social workers and strengthen their capacity to provide effective case management addressing both VAW and VAC. In Iraq, for example, some suggested providing sensitivity training for child protection officers, especially when handling cases related to violence against girls. Building the skills of frontline workers in managing the complexities of intersecting forms of violence was considered essential to improving the quality and consistency of response services.

*“Critical to our team’s development is training on case management related to gender-based violence and the nuanced handling of survivors. Equally crucial is training on general principles of child protection and the provision of child protection services.”* [I2, government actor, Iraq]

Some participants insisted that separating the mothers’ health from child’s needs was impractical when designing and delivering interventions. These views reinforced the argument supported by many participants that assessing maternal safety is essential when providing services to children, and vice versa.

*“I see it like this, from my department and my perspective, that when you work on breastfeeding and in nurseries and when you work with early childhood development […] you cannot detach the child from their mother or their caregiver […] there’s a dynamic between them.”* [L9, government actor, Lebanon]

Finally, participants emphasised the need to strengthen evidence about effective collaborative approaches and increase and sustain funding for data collection, research, and training to support long-term programme implementation.

*“[We need] evidence-based data; we don’t have access to resources that would allow us to do research […] to the level that we want.”* [L5, NGO staff, Lebanon]

## Discussion

This study explored national-level perspectives on collaborative approaches between VAW and VAC services in Iraq, Jordan, and Lebanon by analysing stakeholders’ views on challenges and opportunities for improved collaboration. The findings reflect the vantage point of government and NGO actors engaged in strategic service planning, policy implementation, and inter-agency coordination. They provide valuable insight into policy and systems-level challenges and facilitators (protocols, mandates, legal frameworks, funding) shaping the possibility of collaborative responses.

Important to note is that participants spoke about collaboration to refer to a range of coordination models ranging from data information-sharing, aligned protocols and referral pathways, to co-location of services or same provider, rather than full structural integration. In the study contexts, strengthened referral linkages, joint planning, and shared standards were often seen as more realistic than full institutional merger (which is not necessarily an intended objective), given existing mandates, resource constraints and legal fragmentation.

Participants consistently emphasised the need for a collaborative approach, citing the intersecting and often co-occurring nature of VAW and VAC. The findings resonate with the broader literature on VAW and VAC intersections, which emphasises shared risk factors such as gender inequality and intergenerational cycle of abuse, co-occurrence within households, and the necessity of coordinated responses [11,14,38,39].

The study suggests several opportunities for strengthening collaboration across VAW and VAC systems. A key opportunity lies in working with community and religious leaders, who often hold significant influence in shaping social norms and mediating disputes that may perpetuate violence [40]. While some participants, especially in Lebanon, had reservations on this approach, others reflected on how instrumental such leaders can be discussing sensitive topics, mediating disputes, and creating entry points for addressing both VAW and VAC. This is consistent with emerging evidence, which suggests that engaging faith and religious leaders can contribute to challenging gender and social norms that condone violence, encouraging community support, and influencing legislative changes to support for services that both aim to prevent as well as respond to violence against women and children improving help-seeking. Though impacts are highly contingent on intensive training, efforts to challenge harmful scripts misinterpretations and strong referral links to VAW and VAC support services [40–43].

Maintaining a balance between preserving family values and addressing VAW and VAC is particularly nuanced in the study contexts. While family unity is often prioritised, it is important to note that evidence suggests that couples counselling is not recommended in cases involving ongoing IPV, as it may reinforce existing power imbalances, compromise survivor safety, and offer limited or context-dependent evidence of effectiveness [44]. As some participants noted, interventions perceived as threatening family values may face resistance, even when they are rights-based and evidence-informed. This underscores the tension between prevailing social norms and human rights frameworks in responding to VAW and VAC. The challenge is not reconciliation per se, but reconciliation that occurs in the presence of ongoing risk, as these approaches assume equal bargaining power between parties (a condition rarely met in abusive relationships) or reconciliations that are prioritised over survivor-defined safety goals (including separation) without adequate risk assessment or safeguards [45–47].

Although not a primary finding of our study, several participants emphasised the importance of community-wide involvement, including youth and male engagement in VAW and VAC programmes, to enhance the acceptability of interventions aimed at shifting harmful norms. Current literature suggests that initiatives that engage men and boys in addressing gender, power, and violence can reduce risks of both VAW and VAC by positioning boys and men as allies in prevention [48,49]. Such initiatives engaging men and boys are most effective when combined with parallel support to women and with broader community mobilisation and social norm change efforts, rather than delivered as stand-alone activities [1].

However, three key systemic barriers emerged. First, fragmented and inconsistent legal frameworks, including contradictory laws with differing age thresholds and gaps in protection for adolescent girls, which undermine the creation of survivor-centred approaches [11]. In Iraq, Article 41 of the Penal Code, which permits domestic violence within “certain limits”, continues to hinder protections for women and children [50]. In Jordan, while the National Framework for Family Protection emphasises a multisectoral approach to family violence, societal norms still frame domestic violence as a private matter, discouraging survivors from seeking help [26].

Second, resource and capacity constraints, such as competition for donor funding and siloed operational culture between service sectors, limit the incentives and practical mechanisms for collaboration. For example, in Jordan and Lebanon, gaps in capacity among social workers and law enforcement personnel exacerbate the disconnect between VAC and VAW service streams. These professionals often operate within different conceptual and operational frameworks, leading to siloed responses even in shared service spaces [11]. Additionally, funding competition among NGOs and narrow donor interests often discourage collaborative efforts. Beyond funding shortages, institutional competition and concerns about mandate “ownership” (i.e., the delineation of distinct institutional responsibilities) between women and child protection bodies, and other ministries can further weaken incentives for coordination. Fragmented data systems and differing case definitions also impede joint monitoring and learning across VAW and VAC services, limiting visibility of co-occurrence, missed opportunities for joint responses, and accountability for coordinated pathways.

Third, entrenched cultural and gender norms, privileging family unity over survivor safety and favouring mediation or reconciliation in domestic violence cases, constrain the uptake of integrated interventions and may place survivors at risk. Across the three countries, societal emphasis on preserving family unity often supersedes the safety of women and children, as seen in the preference for reconciliation processes or mediation in cases of domestic violence. This resonates with critiques in the literature on reconciliation processes, including global guidance advising against mediation or reconciliation in IPV cases, which may re-expose survivors to harm [45–47]. Istratii and Ali suggest in their scoping review that responses to IPV in religious communities often intersect with ideas of forgiveness, marital repair, and family preservation. Many faith-based perspectives frame reconciliation as a broader process grounded in ethical accountability, repentance, and the protection of the family unit, which may or may not require continued cohabitation with the perpetrator [40]. In our study settings, where state protection and social welfare systems remain limited, the preference for reconciliation may also reflect pragmatic realities rather than purely ideological commitments to family preservation. The challenge therefore lies in determining how reconciliation processes can mitigate harm and maintain social stability without compromising women’s rights or safety. The need for routine risk assessment, safety planning, and safeguards remains essential when families choose to maintain contact, ensuring that survivor agency and protection considerations guide any dispute-resolution processes.

The study underscores the importance of adopting a collaborative approach to addressing VAW and VAC which can enhance service delivery by reducing duplication, increasing resource efficiency, and ensuring holistic support for survivors [1,10]. Strengthening such approach requires targeted cross-training to frontline workers (from VAW and VAC fields) to build the skills and capacities in recognising intersecting risks, supporting safe disclosure, and navigating referral pathways across sectors. Beyond training, strengthened collaboration requires joint or harmonised policies that explicitly link VAW and VAC, aligned SOPs for identification, case management and referral, and sustained multisectoral coordination platforms that bring together VAW and VAC actors for planning, oversight, and accountability. Community-based prevention and social and behavioral change interventions offer important entry points for reshaping harmful norms and increasing the acceptability of collaboration, aligning with gender-transformative approaches seeking to challenge structural inequalities perpetuating violence [39,51]. However, it is important to note that local resistance to VAW programmes can be shaped by perceptions of Western ideological imposition and association of gender-equality discourse with foreign cultural agendas. Thus, context-sensitive approaches that engage with locally grounded ethical and social frameworks are necessary to address violence in ways that maintain legitimacy and community trust [40]. Our findings also suggest that, in these contexts, child protection or family protection can sometimes provide a more acceptable way to open conversations about VAW, but this needs to be done carefully so that women’s own safety, rights and support needs are not lost within a broader family framing.

Collaboration between VAW and VAC providers can be further hindered by uneven organisational capacity, differing mandates, and the risk of overburdening already under-resourced services. In such conditions, parallel but complementary interventions may be more feasible and effective than fully integrated services, allowing providers to maintain sector-specific expertise while addressing intersecting risks, particularly when anchored in shared frameworks, robust referral mechanisms, and joint monitoring and accountability arrangements that support better integrated survivor journeys.

Our study offers several contributions that go beyond reaffirming well-documented barriers to collaborative efforts within the VAC and VAW sectors. First, it provides cross-country comparative evidence from Iraq, Jordan, and Lebanon, showcasing how coordination between VAW and VAC sectors is shaped by distinct institutional and socio-political contexts within the region. Second, it offers practical insight by identifying specific mechanisms that enable or hinder collaboration, including joint case management structures, integration of referral pathways, and the strategic framing of VAC as an entry point for addressing VAW and broader family violence.

### Limitations

Several limitations should be considered when interpreting the findings of this study. The purposive and relatively small sample consisted primarily of higher-level stakeholders engaged in strategic planning, policymaking, or coordination of VAW and VAC services. As participant recruitment and validation processes were supported by UNICEF country offices, the perspectives represented may be more reflective of stakeholders engaged in or connected to existing coordination efforts. This may have also meant that the findings gave more weight to formal coordination structures and policy-level perspectives, while the views of smaller, community-based or less well-connected organisations may have been less visible. The study could not assess women, children and families’ experiences of collaboration, or whether coordinated approaches improve their access, safety or quality of care, as these actors were not included. As a result, the perspectives, experiences, and local innovation of frontline practitioners, as well as the voices of service users themselves, are not directly represented. This limits our ability to capture the full complexity of collaborative practices in service settings, particularly in rural or resource-constrained contexts, underscoring the need for an expanded sample size. While these insights expose crucial structural barriers and institutional opportunities for collaboration from national-level stakeholders, they may omit operational challenges and adaptive strategies developed at the community level or within implementing agencies.

While participants may not have represented the full spectrum of VAW & VAC actors, we believe that those who participated gave sufficient information on existing services, barriers and opportunities for collaboration. Future research should address these gaps by including the lived experiences of frontline personnel and service users, women and their families utilising mixed-methods and participatory approaches, and incorporating longitudinal evaluations to assess the practical impacts and sustainability of coordination efforts in diverse local contexts. Ideally, this would pair top-down policy alignment with bottom-up engagement, ensuring that the voices of staff, community actors, and survivors inform feasible, context-sensitive solutions. Although boys were represented in some of the included documents on VAC, the study did not specifically examine gender differences when it comes to VAW and VAC sectoral collaboration. Finally, it is important to note that data collection took place in 2023, prior to the escalation of Israeli attacks on Lebanon, the formation of the Lebanese new government, and subsequent shifts in the global aid landscape. As a result, the findings may not fully capture more recent political, funding, and policy dynamics and should be interpreted accordingly.

## Conclusion

Our study highlights both the extent and limitations of current VAW and VAC collaboration efforts in Iraq, Jordan, and Lebanon. While Lebanon and Jordan demonstrate emerging policy-level commitments through multi-sectoral family protection frameworks, coordinated programming remains limited across all three countries. Our findings suggest that key opportunities to strengthen collaboration efforts in resource-constrained and politically unstable settings may not be defined by full integration, but through pragmatic, context-adapted mechanisms. These include functional referral pathways, shared or coordinated case management, and strategic entry points, such as child protection, which enable engagement with VAW in contexts where direct approaches may be hindered. Strengthening collaboration requires moving beyond formal policy alignment toward investments in institutional capacity, cross-sector training, and culturally informed approaches that account for prevailing social norms. Additionally, rather than requiring full institutional integration, effective collaboration in resource-constrained and politically fragmented settings is more likely to be achieved through context-sensitive coordination mechanisms (such as strengthened referral pathways, shared standards, joint planning, and information sharing) that build on existing mandates and institutional arrangements.The study also calls for generating context-specific evidence on which approaches most effectively, safely, and equitably address both VAW and VAC outcomes.

## Supporting information

S1 Checklist Inclusivity in global research. (PDF)
